# Receptor-mediated mitophagy: a new target of neurodegenerative diseases

**DOI:** 10.3389/fneur.2025.1665315

**Published:** 2025-11-18

**Authors:** Junlan Yang, Fuquan Yang, Guiyan Chen, Ming Liu, Shiqing Yuan, Tian-e Zhang

**Affiliations:** 1 School of Basic Medicine, Chengdu University of Traditional Chinese Medicine, Chengdu, Sichuan, China; 2 Guangyuan Hospital of Traditional Chinese Medicine, Guangyuan, Sichuan, China; 3 Chengdu First People's Hospital, Chengdu, Sichuan, China

**Keywords:** neurodegenerative diseases, PINK1/Parkin-independent mitophagy, autophagy receptors, mitochondria, mitophagy, mitochondrial dysfunction

## Abstract

Neurodegenerative diseases are a category of neurological conditions with high prevalence that pose major treatment challenges. Common pathologies involve protein accumulation and mitochondrial damage. Mitophagy maintains cellular homeostasis by removing defective mitochondria, which are associated with the pathogenesis of neurodegenerative diseases. Although the ubiquitin-dependent mitophagy mediated by the PINK1–Parkin pathway has been extensively studied, growing evidence indicates that receptor-mediated mitophagy plays a crucial compensatory role in neurons, particularly when the PINK1–Parkin pathway is impaired. This review focuses on the emerging field of receptor-mediated mitophagy, systematically elaborating its role as a key homeostatic mechanism operating independently of the canonical PINK1/Parkin pathway. It provides a focused analysis of the specific functions and activation mechanisms of key receptors—including BNIP3, NIX, FUNDC1, and AMBRA1—in models of Alzheimer’s disease, Parkinson’s disease, and amyotrophic lateral sclerosis. Furthermore, this review explores the clinical potential of targeting these specific receptors for precise intervention, aiming to provide a new theoretical foundation and direction for developing therapeutic strategies against neurodegenerative diseases.

## 1 Introduction

Neurodegenerative diseases (NDDs) are a group of disorders characterized by progressive loss of neuronal structure and function in the central nervous system. Pathological features typically include a sustained decrease in neurons, abnormal proliferation of glial cells, and activation of neuroinflammatory responses. As the disease progresses, patients generally experience significant motor dysfunction, cognitive decline, and sensory loss, which are progressive and irreversible (1). It is indisputable that aging, as a major risk factor for most NDDs, is because neurons in the brain are susceptible to aging-induced changes, highlighting one of the key factors in the aging process: mitochondrial function (2). Mitochondria act as the “energy factories” of the cell, producing Adenosine Triphosphate (ATP) through oxidative phosphorylation via the electron transport chain. This is the main energy source for the cell and is required by neurons, which have a higher energy demand (3). Different parts of a neuron have varying energy demands, which require a fully functional mitochondrial network to sustain their complex structures and functions—including synaptic transmission, long-distance axonal transport, and mitochondrial fusion and fission (4). Therefore, any factor that disrupts the balance of the mitochondrial quality control system can trigger neuronal damage or even cell death.

Mitophagy plays a critical role in maintaining neuronal health among the various mitochondrial quality control mechanisms. The focus in this field has traditionally been on the canonical ubiquitin-dependent pathway mediated by PINK1 (PTEN-induced putative kinase 1) and Parkin (Parkinson protein 2). This pathway is collectively recognized as the central mechanism for responding to mitochondrial damage, and its dysfunction is well-established in the pathogenesis of numerous neurodegenerative diseases (5).

However, it has been demonstrated that there exists a form of “receptor-mediated mitophagy” pathways that are not dependent on PINK1-Parkin in the context of pathologies in which PINK1 or Parkin expression is absent or functionally impaired, as well as in neurodegenerative disorders more generally (6). These pathways are directly triggered by specific receptor proteins, which recruit autophagy components with greater directness and superior efficiency. While existing literature has extensively described the general mechanisms of mitophagy, comprehensive summaries of receptor-mediated mitophagy remain inadequate, often relegating it to a secondary or complementary role. This review systematically summarizes recent advances in the role of receptor-mediated mitophagy in neurodegenerative diseases. We will emphasize that this autophagic pathway is not merely a simple backup for the canonical pathway but serves as a crucial and independent clearance mechanism under specific conditions. We aim to provide a novel perspective for understanding NDDs pathogenesis and to establish a theoretical foundation for developing precise therapies that target specific receptor pathways.

## 2 Quality control of mitochondria

**Figure 1:**
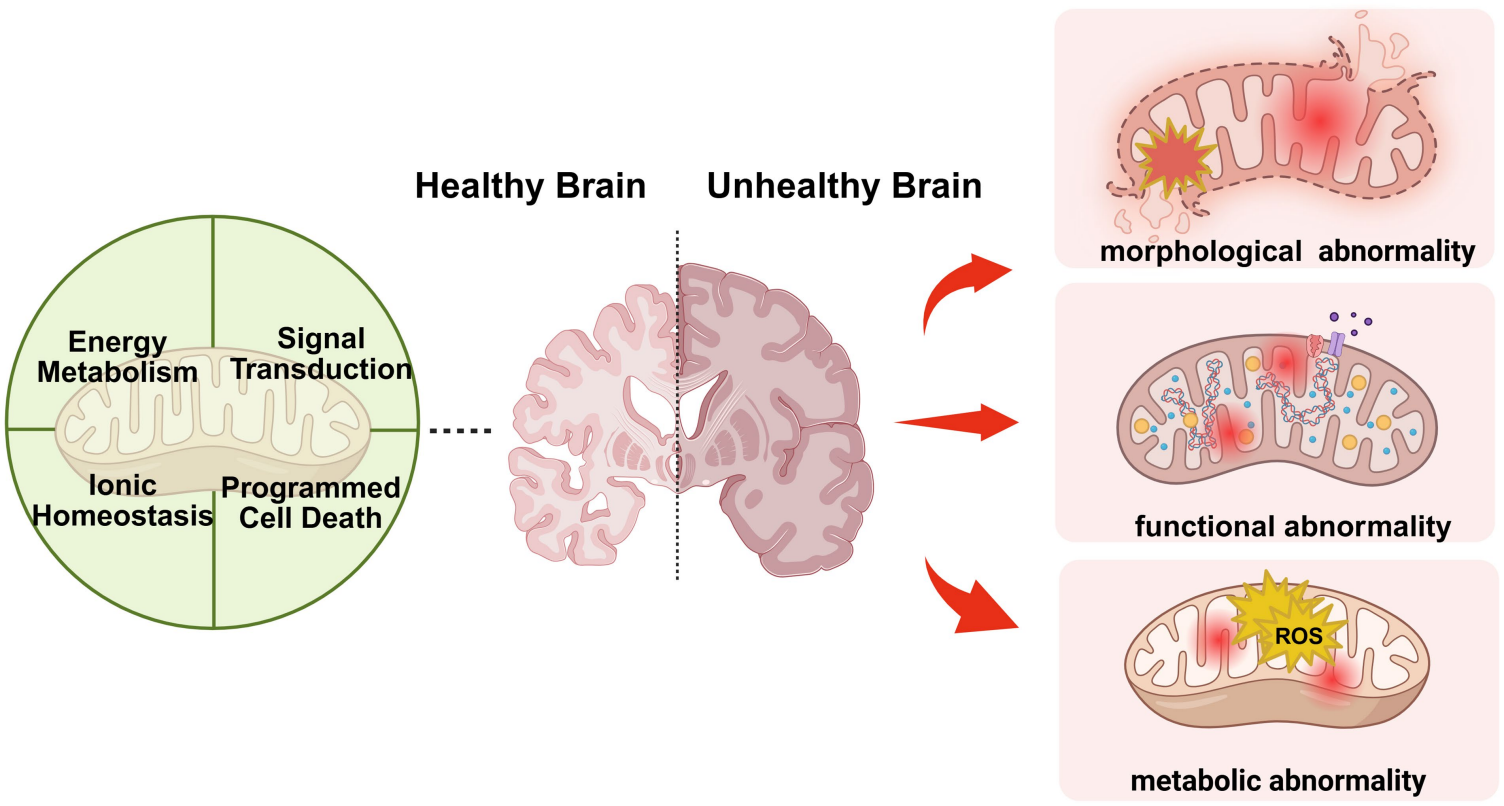
The relationship between mitochondria and neurodegenerative diseases (created with BioRender.com).

Different endogenous and exogenous stimuli may cause mitochondrial dysfunction, and aggregation of abnormal mitochondria in the neurons can lead to imbalances in energy metabolism, impaired signaling, dysregulation of calcium homeostasis, activation of neuroinflammation, and other pathologies. Fortunately, mitochondria are self-regulating organelles that can sense abnormal states within their bodies and regulate their stabilization through a variety of regulatory mechanisms, including mitophagy, mitochondrial fission and fusion, mitochondria-derived vesicles, proteasomal degradation, and exosome release to remove damaged proteins and mitochondria (7–9). Among these mechanisms, mitophagy has received particular attention in recent years. Mitophagy is a subtype of macroautophagy that selectively removes damaged mitochondria via lysosomal degradation. Mitophagy is initiated when other quality control mechanisms have limited processing capacity or when mitochondria need to be removed due to cellular metabolic demands. Mitophagy plays an important role in normal cellular function and influences disease development, and the molecular mechanisms involved in mitophagy have been characterized in a variety of diseases, including aging (10), neurodegenerative diseases (11), and cancer (12). In neurodegenerative diseases, defective mitophagy is thought to be responsible for the accumulation of damaged mitochondria in neurons and leads to neuronal dysfunction and even death, and thus, mitophagy is being explored as a therapeutic target for neurodegenerative diseases (Figure 1).

## 3 Mitophagy pathways

**Figure 2:**
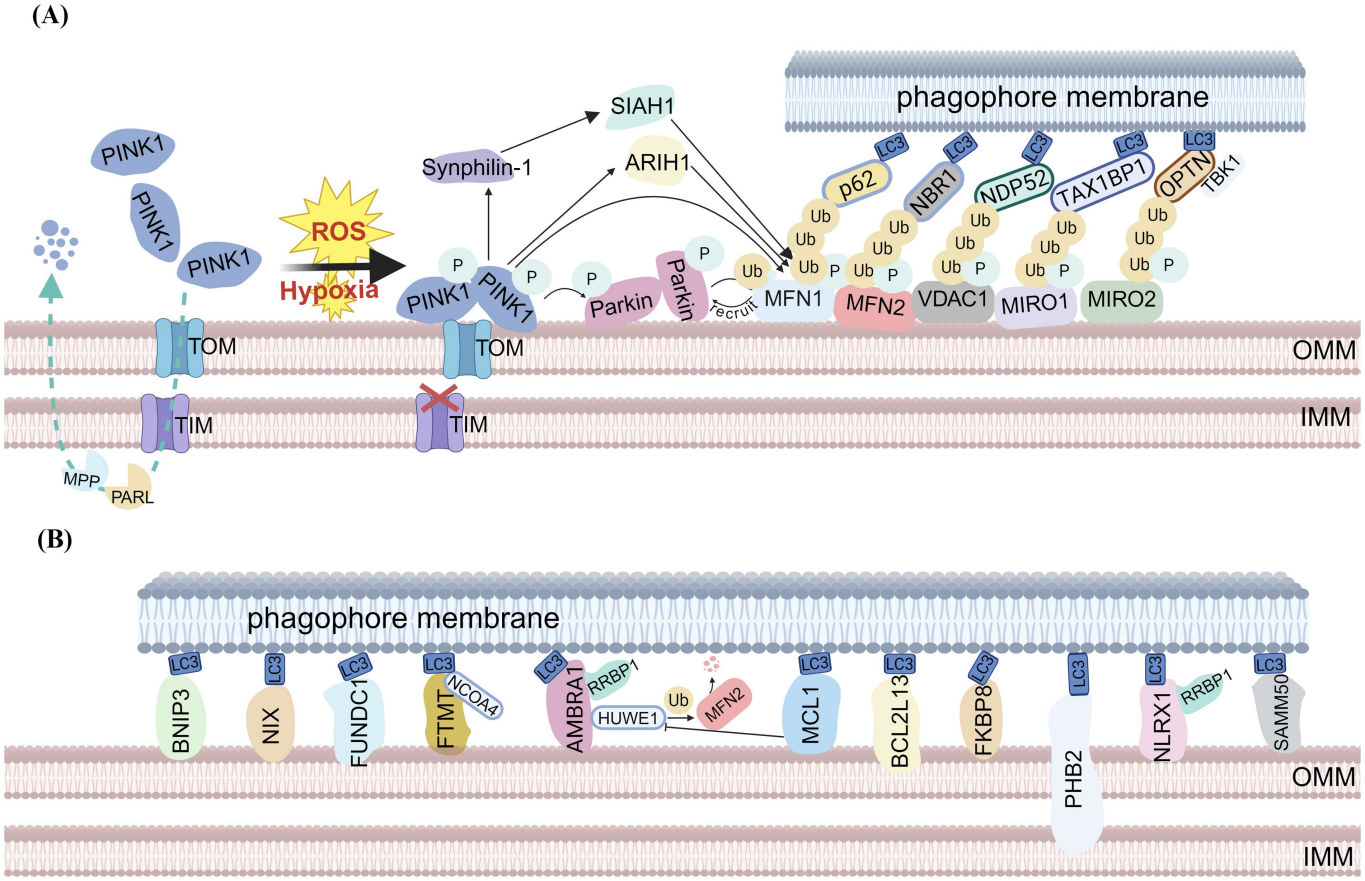
Overview of the mitophagy pathway. **(A)** PINK1-Parkin-mediated mitophagy. Under normal conditions, newly synthesized PINK1 translcates into IMM via the TOM/TIM complexes. PARL cleaves PINK1 at its transmembrane domains, and the cleaved PINK1 is subsequently degraded rapidly by the proteasome, and PINK1 maintains low protein levels. When mitochondria are damaged, PINK1 is unable to translocate to the IMM and is activated at the OMM. Accumulated PINK1 recruits and phosphorylates Parkin, which transforms to an activated E3 ubiquitin ligase through a series of structural remodeling cascade reactions to ubiquitinate many OMM proteins. These ubiquitinated substrates undergo further PINK1-dependent phosphorylation, creating feedforward amplification that recruits additional Parkin to mitochondria. This cascade enhances ubiquitin chain formation and recruits autophagy adaptors, leading to mitophagy. **(B)** Receptor-mediated mitophagy. Mitophagy receptors such as BNIP3, NIX, FUNDC1, FTMT, AMBRA1, MCL-1, BCL2L13, FKBP8, PHB2, NLRX1, and SAMM50 can directly bind to LC3 and GABARAP-family members at the phagophore membrane (created with BioRender.com).

Mitophagy was first proposed by Lemasters in 2005 (13). With advancing research in this field, the regulatory mechanisms of mitophagy have been increasingly elucidated. The mitophagy pathways mainly consist of ubiquitin (Ub)-dependent pathways and Ub-independent pathways (14). The ubiquitin-dependent pathway includes not only the classical PINK1-Parkin mediated mitophagy but also mitophagy mediated by other E3 ubiquitin ligases such as Siah E3 ubiquitin protein ligase 1 (SIAH1) (15), mitochondrial E3 ubiquitin protein ligase 1 (MUL1) (16), HECT, UBA and WWE domain containing E3 ubiquitin protein ligase 1 (HUWE1) (17), and Glycoprotein 78 (GP78), which promote mitophagy by ubiquitinating damaged mitochondrial proteins (18). Ub-independent pathways can be further categorized into receptor-mediated and lipid-mediated mitophagy. The PINK1-Parkin mitophagy pathway, also called classical mitophagy, is the most widely studied mitochondrial quality control way. Its core mechanism relies on the synergistic action of PINK1 and the E3 ubiquitin ligase Parkin. PINK1, a mitochondria-targeted serine/threonine kinase encoded by the PARK6 gene, triggers subsequent Parkin-dependent light chain 3 (LC3) recruitment (19). Under physiological conditions, PINK1 maintains low protein levels through continuous translocation to the inner mitochondrial membrane (IMM) via the TOM/TIM translocase complex, followed by proteolytic processing and degradation (20). When mitochondria are damaged, PINK1 is unable to translocate to IMM and is activated at the outer mitochondrial membrane (OMM) (21). Accumulated PINK1 recruits and phosphorylates Parkin, which transforms from a self-repressing dormant enzyme to an activated E3 ubiquitin ligase through a series of structural remodeling cascade reactions to ubiquitinate many OMM proteins, such as mitofusins 1/2 (MFN1/2). These ubiquitinated substrates undergo further PINK1-dependent phosphorylation, creating feedforward amplification that recruits additional Parkin to mitochondria. This cascade enhances ubiquitin chain formation and recruits autophagy adaptors. Autophagy adaptors are a class of proteins characterized by the presence of an LC3-interacting regions (LIRs), which mainly includes sequestosome 1(P62) (22), neighbor of BRCA1 gene 1 (NBR1) (23), nuclear dot protein 52 (NDP52), human T-cell leukemia virus type I binding protein 1 (TAX1BP1) (24) and optineurin (OPTN). Next, these adaptors bind to LC3 on autophagophore membranes via LIRs, facilitating engulfment of depolarized mitochondria into autophagosomes. The completed autophagosomes are ultimately degraded by lysosomes (14). Notably, TANK-binding kinase 1 (TBK1) phosphorylates OPTN in this process, serving to amplify the signal and thereby enhance mitophagy (25). (Figure 2A). In addition to ubiquitin-dependent mitophagy, specific mitochondrial proteins act as mitophagy receptors. These receptors directly target dysfunctional mitochondria to the phagophore for degradation via protein–protein interactions, bypassing ubiquitination. Key examples include BCL2-interacting protein 3 (BNIP3), NIP3-like protein X (NIX/BNIP3L), FUN14 domain-containing protein 1 (FUNDC1), as well as Autophagy/Beclin-1 Regulator-1 (AMBRA1), among others. All these receptors contain a conserved LC3-interacting region (LIR) motif that directly binds to Atg8-family proteins (e.g., LC3) on the phagophore membrane, thereby recruiting damaged mitochondria to nascent autophagosomes (26) (Figure 2B). Critically, these receptors operate independently of both PINK1-Parkin recruitment and ubiquitination, they have been considered as potential targets for neurodegenerative diseases. While the mechanisms underlying general mitophagy are well characterized, how these receptor-mediated pathways achieve spatiotemporal coordination with parallel pathways requires further investigation.

## 4 Receptor-mediated mitophagy

### 4.1 Hypoxia-dependent receptors

**Figure 3:**
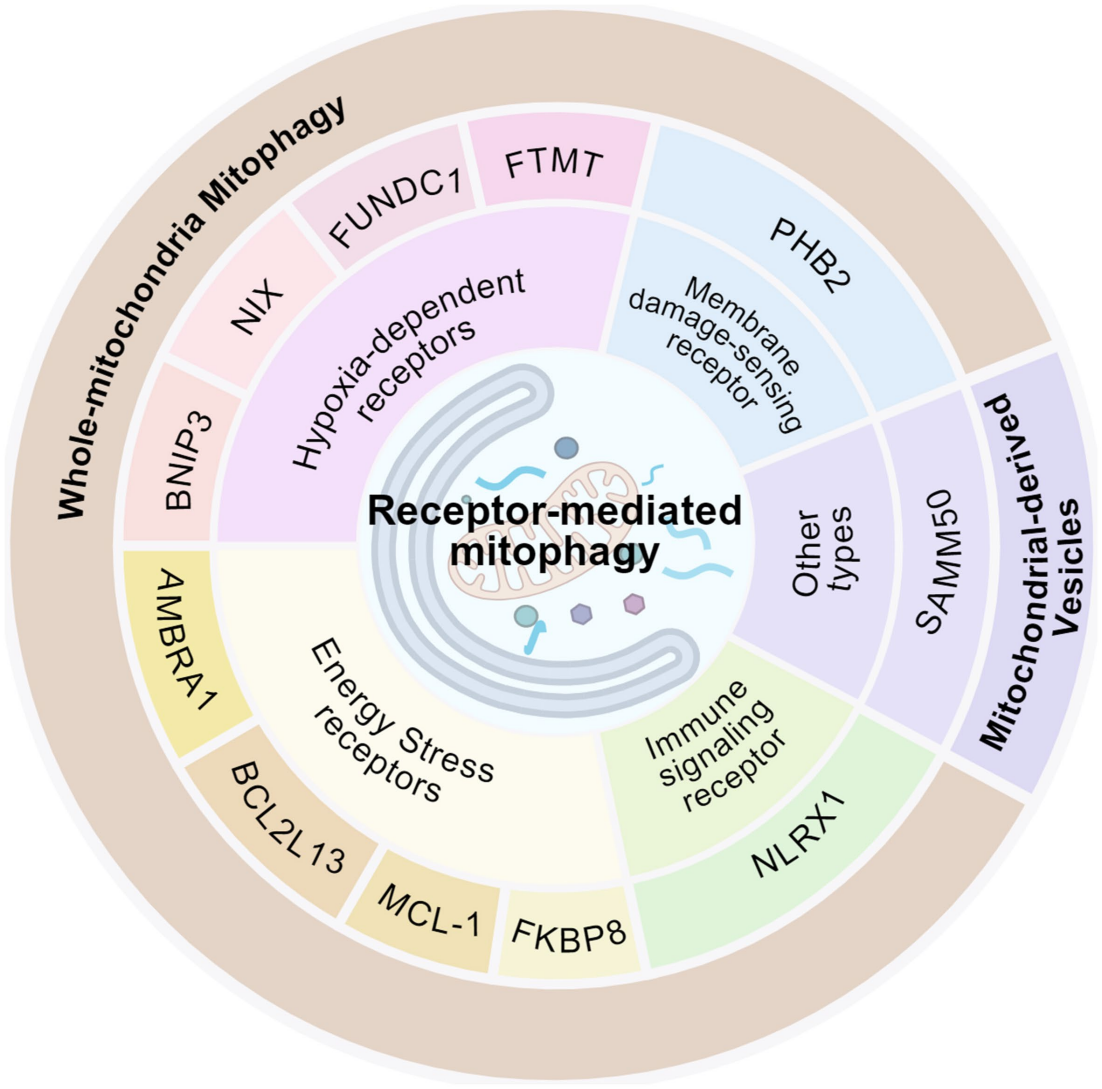
Schematic diagram of mitophagy receptors classification (created with BioRender.com).

These receptors are characterized by their core role in hypoxia-activated mitophagy, directly sensing low oxygen to trigger the process via varied mechanisms for metabolic adaptation (Figure 3). Their activation strategies, though diverse, ultimately converge on a parallel regulatory approach at both the transcriptional and translational levels. This integrated strategy ensures that cells can effectively launch mitochondrial quality control and sustain metabolic stability in response to both acute and prolonged oxygen deprivation.

#### 4.1.1 BNIP3 and NIX

**Figure 4:**
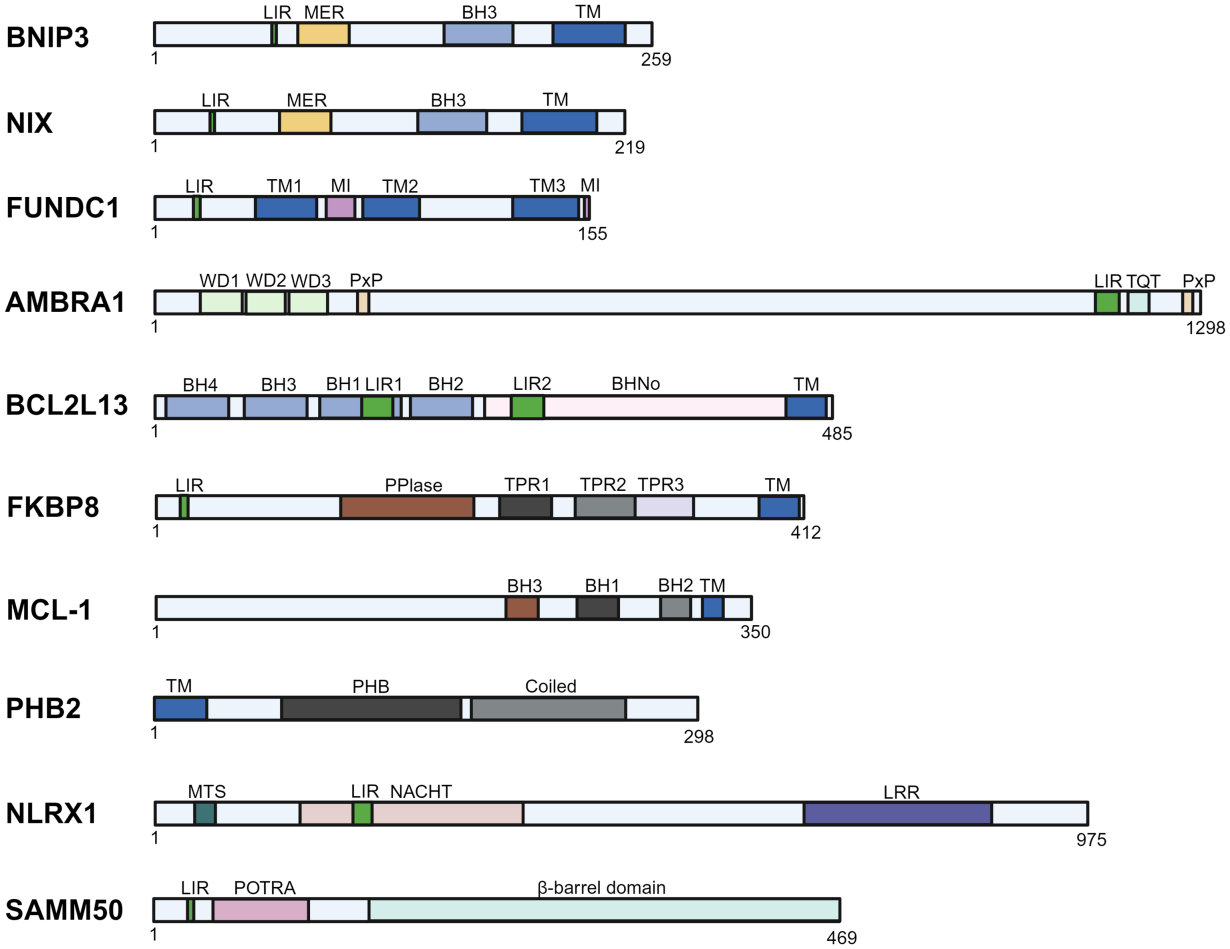
Schematic representation of the domains of mitophagy receptors (created with BioRender.com).

BCL2/adenovirus E1B 19-kDa interacting protein 3 (BNIP3) belongs to the BH3-only subfamily of the Bcl-2 protein family, and it regulates the permeability state of OMM. The structure of BNIP3 comprises a large complex N-terminal region. This N-terminal region contains a WXXL motif, which serves as its LIR. Via this motif, BNIP3 binds to LC3 and induces mitophagy (Figure 4) (27).

NIX is a multifunctional BH3-only protein of the Bcl-2 family localized to the OMM. It shares 65% identity with BNIP3 in amino acid sequence (28, 29). The LIR motif of NIX consists of four amino acid motifs that are essential for interaction with LC3 and GABA type A receptor-associated protein (GABARAP), and deletion of the LIR motifs in NIX leads to failure of mitophagy (Figure 4) (30). The role of NIX in mitophagy was initially identified in Nix-knockout mice, which exhibit anemia due to impaired mitophagy, resulting in dysregulated reticulocyte maturation and massive mitochondrial accumulation (31, 32).

Upon the onset of hypoxia, BNIP3 and NIX dimerize and anchor to the OMM through their TM domains; BNIP3 homodimerization is critical for mitophagy, and failure of homodimer formation impairs this process (33). Studies show that besides binding LC3 to induce mitophagy, these receptors enhance Parkin-mediated mitophagy and functionally compensate for Parkin deficiency. Specifically, BNIP3 interacts with PINK1 to inhibit the protein degradation of PINK1, which then participates in ubiquitin-dependent mitophagy by promoting the translocation of Parkin to mitochondria. Moreover, NIX is a Parkin substrate; when ubiquitinated by Parkin, NIX recruits the selective autophagy adaptor NBR1 and LC3/GABARAP to mitochondria to initiate perimitochondrial phagophore assembly (34). NIX also contributes to Parkin accumulation on damaged mitochondria (35). Furthermore, it has been shown that mitophagy clearance in axonal mitochondria requires NIX but not Parkin. This study supports different mechanisms between NIX and Parkin (36).

Studies primarily focus on hypoxia-inducible factor 1-alpha (HIF-1α) activating BNIP3 transcription during hypoxia, upregulating its expression. Consequently, BNIP3 and NIX are classified as hypoxia-specific mitophagy factors. Besides, some other regulators, such as transcription factors, growth factors, or cellular stresses, are also involved in NIX regulation, while BNIP3 and NIX expression may also be regulated by some microRNAs (35). Phosphorylation represents the most studied mechanism underlying BNIP3/NIX-mediated mitophagy. BNIP3 phosphorylation at Ser17/24 enhances LC3B affinity and increases autophagosome recruitment to mitochondria. c-Jun N-terminal kinase 1/2 (JNK1/2) phosphorylates BNIP3 at Ser60/Thr66 under hypoxia, stabilizing BNIP3 and facilitating direct LC3 binding to drive mitophagy. However, severe hypoxia inactivates JNK1/2, inducing BNIP3 dephosphorylation and suppressing mitophagy (37). NIX phosphorylation at Ser34/35 activates mitophagy via its LIR motif, whereas Ser212 phosphorylation inhibits dimerization, suggesting that phosphorylation of different serine residues would have opposite results. Ubiquitination provides another regulatory mechanism. Ubiquitinated NIX recruits adaptor NBR1 in the autophagosomes for mitophagy (34). Recently, the mitochondrial outer membrane protein FBXL4 has been identified, which recognizes and ubiquitinates BNIP3 and NIX, thereby promoting their degradation and suppressing mitophagy (38). Meanwhile, PPTC7 plays a key role as a connector molecule in assembling the BNIP3/NIX-PPTC7-SCFFBXL4 complex to degrade BNIP3 and NIX and form a homeostatic regulatory loop, which helps to maintain mitochondrial number and function, and prevents cellular energy disruption from excessive mitophagy (39). It is clear that hypoxia is not the only inducer of activation of this pathway, but the specific mechanisms by which BNIP3 and NIX regulate mitophagy have not been fully elucidated and require further follow-up studies.

#### 4.1.2 FUNDC1

FUNDC1 is an OMM-localized mitophagy receptor protein that belongs to the paralogous subfamily of FUN14 domain-containing proteins (40). Its structure comprises a cytoplasmic N-terminus, a C-terminal region, and an OMM transmembrane region, where the N-terminus is exposed to the cytosol and the C-terminus extends into the intermembrane space. The N-terminus contains a typical LIR motif: the Y(18)xxL (21) sequence, which interacts with Atg8 family proteins to induce mitophagy (Figure 4) (41). It has been reported that during hypoxia, FUNDC1 is subject to transcriptional-level gene upregulation to enhance mitophagy (42). The regulation, however, is largely governed by rapid post-translational modifications. Under hypoxic stress, FUNDC1 undergoes dephosphorylation at Ser13 and Tyr18 sites, activating its mediated mitophagy. A key representative regulator is the PGAM family member 5 (PGAM5), which promotes dephosphorylation of FUNDC1 at Ser13 in its multimeric form, thereby serving as a positive regulator of mitophagy (43). Meanwhile, during energy depletion, the mitophagy initiation molecule unc-51-like autophagy activating kinase 1 (ULK1) is activated and recruited to mitochondria, phosphorylates FUNDC1 at Ser17, and promotes mitophagy (44, 45). Studies indicate that there may be crosstalk between ubiquitin-dependent autophagy and mitophagy, and that Membrane-associated ring-CH-Type finger 5 (MARCH5), an E3 ubiquitin ligase, is an important regulator of FUNDC1-mediated mitophagy. During early hypoxia, MARCH5 homo-oligomers dissociate into monomers; subsequently, MARCH5 binds Lys119 of FUNDC1, promoting FUNDC1 ubiquitination and degradation to suppress mitophagy and prevent inappropriate clearance of undamaged mitochondria (46). Whether FUNDC1 has additional modifications remains to be further investigated, and how multiple modifications can be coordinated to regulate the state of FUNDC1 will be the focus of future studies.

#### 4.1.3 FTMT

Mitochondrial ferritin (FTMT) is important for intracellular iron homeostasis, antioxidant defense, inflammatory regulation, and multiple pathological processes. This protein is primarily localized to mitochondria, but under stress conditions such as hypoxia or inflammation, it can be cleaved by thrombin and translocate to the cytoplasm, demonstrating dynamic subcellular distribution (47). Current research indicates that FTMT’s involvement in mitophagy is closely linked to its role in regulating HIF-1α stability and its interaction with nuclear receptor coactivator 4 (NCOA4). Under hypoxic conditions, HIF-1α upregulates FTMT expression, FTMT interacts with NCOA4, and increases the co-localization of FTMT with LC3, thereby promoting mitophagy (48, 49). In addition, the expression of NCOA4 was significantly reduced under hypoxic conditions. This reduction led to the restriction of the ferritin degradation pathway, increasing the accumulation of FTMT, and the excess free iron catalyzed the ROS generation, causing mitochondrial oxidative damage and triggering mitophagy (50). The mechanism by which FTMT mediates mitophagy is not fully understood, and in particular, how changes in its localization in different cell types and stress contexts affect mitophagy initiation remain controversial. For example, the translocation of FTMT to the cytoplasm after thrombin cleavage under hypoxic conditions may alter its original autophagy-related functions.

### 4.2 Energy stress receptors

The activity of these receptors is regulated by energy stress pathways and primarily responds to cellular energy metabolic imbalances. Under stress conditions, their activity is significantly enhanced, thereby promoting large-scale clearance of severely damaged mitochondria and preventing cellular apoptosis.

#### 4.2.1 AMBRA1

Autophagy and Beclin1 Regulator1 (AMBRA1) is a multifunctional scaffolding protein. It contains three and a half WD40 repeat sequences at both its N-terminal and C-terminal, which are joined and folded into a *β*-propeller structure. Between the two WD40 structural domains, there are 650 amino acids, as well as 250 amino acids at the end of the protein, constituting an intrinsically disordered region (51). This region is essential for AMBRA1 regulation. The LIR region is located at its C-terminus (Figure 4). In the PINK1-Parkin independent pathway, AMBRA1 binds to LC3B via its LIR motif and mediates the wrapping of mitochondria by autophagosomes. Its LIR motif activity is regulated by CHUK/IKKα kinase phosphorylation (17). A variety of post-translational modifications and signaling pathways regulate AMBRA1 activity. In the resting state, its activity is suppressed by phosphorylation of the Ser52 site by MTOR or by binding to BCL2 (52). Under stress conditions, ULK1 phosphorylates AMBRA1 and translocates to the mitochondria-associated ER membrane (MAM) to activate mitophagy (53). In addition, AMBRA1 is recruited into the OMM during mitochondrial damage, prevents the translocation of PINK1 to the mitochondrial matrix, and aggregates at the OMM by binding to the TOM complex and ATAD3A, and enhances its stability, preventing it from being degraded by mitochondrial proteases, which in turn initiates the Parkin-mediated mitochondrial ubiquitination (54). Recent studies have shown that AMBRA1 stability is regulated by CRL4-mediated ubiquitination degradation as a means of limiting excessive mitophagy, but the exact regulatory mechanism is unclear.

#### 4.2.2 BCL2L13

Bcl2 like 13 (BCL2L13) is a mitochondrial-localized protein belonging to the Bcl-2 protein family. Discovered two decades ago as a functional homolog of yeast’s classical mitophagy receptor Atg32, it anchors to the mitochondrial outer membrane and is expressed in various cells and tissues. BCL2L13 contains four BH domains (BH1-4) and a BHNo region, followed by a transmembrane (TM) domain (55). Uniquely, both the BH and BHNo regions contain a WXXL/I motif (LIR1/2), yet mutation of the motif exclusively in the BHNo region impairs its mitophagic function, indicating that this WXXL/I motif (LIR2, residues 273–276) serves as the LC3-interacting domain (Figure 4). BCL2L13, as a Parkin-independent mitophagy receptor, mediates mitophagy via its unique LIR2 motif, which interacts with LC3, while its mitophagic activity is regulated by Ser272 phosphorylation. PRKAA2/AMPKα2 phosphorylates BCL2L13 at Ser272 (56). In addition, Studies demonstrate that the LIR domain in human BCL2L13 preferentially binds LC3C, GABARAP, and GABARAP-L1 through the ULK1 complex, indicating BCL2L13 exhibits selectivity in mitophagy mediation (57). The physiological significance of this selective binding and the differences in the efficiency and specificity of mitophagy resulting from it in different cell types or tissues are important directions for future research. Despite the functional similarities between BCL2L13 and receptors such as BNIP3, its unique structural basis (e.g., intact BH1-4 domain) and the property of not forming heterodimers with other classical BCL2 family members make it play an irreplaceable role in the regulation of mitochondrial homeostasis, and make it a highly promising target for disease intervention.

#### 4.2.3 MCL-1

Myeloid cell leukemia-1 (MCL-1) is a multifunctional cellular regulatory protein localized to the OMM, notable within the Bcl-2 family for its significant anti-apoptotic activity. MCL-1, like BCL-2, is an anti-apoptotic member of this family and regulates cell viability by maintaining OMM integrity. Early work by Hollville et al. demonstrated that MCL-1 prevents the degradation of damaged mitochondria by suppressing Parkin translocation to OMM (58). Follow-up studies revealed that MCL-1 acts as a mitophagy receptor in a PINK1-Parkin independent pathway, which interacts with LC3A and GABARAP on autophagosomes via the LIR motif to drive mitophagy. Notably, the MCL-1 C-terminus contains three LIR motifs (Figure 4) (59). Co-localization of mitochondria with LC3 is significantly reduced only when these three motifs are simultaneously mutated, but the relative contribution of each LIR under physiological conditions and its regulatory mechanism remain unclear. Moreover, Moyzis et al. demonstrate that MCL-1 exhibits a dual role in autophagy regulation. When nutrients are limited, MCL-1 preserves cellular function by shielding healthy mitochondria from non-selective autophagy; under stress, MCL-1 directly binds to LC3 and promotes the degradation of depolarized mitochondria by synergizing with BNIP3 (59). In addition, MCL-1 also participates in a broader regulatory network, indirectly regulating AMBRA1 activity and the mitophagy process by restricting the recruitment of the E3 ligase HUWE1 to the mitochondria, thus realizing the fine regulation of the mitochondrial mitophagy process (60). These synergistic effects highlight MCL-1 as a key node in the mitophagy network, but how it prioritizes reciprocal partners in different cell types or pathological contexts, and the specific contributions of these reciprocal interactions in physiology and disease, remain gaping points in current research.

#### 4.2.4 FKBP8

FK506-binding protein 8 (FKBP8), also known as FKBP38, is a unique member of the FK506-binding protein (FKBP) family, anchored in the OMM, and a multifunctional junction protein with anti-apoptotic activity. The FKBP8 N-terminal region contains the classical LIR motif, while the C-terminal region contains a TM domain (Figure 4) (61). Under stress conditions, FKBP8 binds to LC3A via its N-terminal LIR sequence to mediate mitophagy, parallel to the PINK1-Parkin pathway. Studies have confirmed that in starvation-induced autophagy, FKBP8 interacts with BECN1 in the VPS34 lipid kinase complex, enhancing the complex’s activity to promote the production of phosphatidylinositol-3-phosphate (PI3P), which initiates mitophagy (62). Interestingly, when autophagosomes encapsulate damaged mitochondria, FKBP8 escapes from acidifying mitochondria to the endoplasmic reticulum (ER) to avoid removal (63). Currently, the precise mechanism underlying FKBP8 escape translocation during mitophagy degradation requires further investigation. Studies demonstrate that abnormally phosphorylated tau protein (p-tau) interferes with this dynamic process of FKBP8, leading to impaired function in autophagy and inhibiting oxidative stress-induced mitophagy by affecting the expression and dynamics of FKBP8 (64). It is noteworthy that FKBP8 also binds to FUNDC1 under hypoxic stress. However, the exact function of this interaction in mitophagy remains elusive.

### 4.3 Membrane damage-sensing receptor

Membrane damage-sensing receptor serve as sensor that respond to structural damage in mitochondria. Upon irreversible, severe damage to the mitochondrial membrane, they recognize the impairment and initiate the autophagic program to remove the completely dysfunctional organelles.

#### 4.3.1 PHB2

Prohibitin 2 (PHB2), a mitophagy receptor protein, resides primarily in the mitochondrial inner membrane (IMM), with an N-terminal transmembrane region and a C-terminal loop region (Figure 4). PHB2 typically binds to PHB1, which is another member of the PHB family, through the C-terminal loop region to form a heterodimer and promote mitophagy (65, 66). Mitochondrial outer membrane rupture exposes PHB2 to the cytosol, enabling mitophagy mediation through binding between its LIR motif and LC3-II at the IMM. Mutation of this domain blocks PHB2-LC3 interaction and suppresses mitophagy. Moreover, PHB2 plays a key role in mitophagy by regulating the PINK1-Parkin pathway, and its activity is negatively regulated by presenilin-associated rhomboid-like protein (PARL). At homeostasis, newly synthesized PINK1 translocates into IMM via the TOM/TIM complexes. PARL, an inner membrane protease, cleaves PINK1 at its transmembrane domains, and the cleaved PINK1 is subsequently degraded rapidly by the proteasome. This process prevents the accumulation of full-length PINK1 at the OMM to activate mitophagy. The process of PHB2 mediating the stable expression of PINK1 relies on the precise regulation of the PARL-PGAM5-PINK1 axis: the binding of PHB2 to PGAM5 effectively blocks the inhibitory effect of PARL on PGAM5 activity and thus maintains the stable state of PINK1. Notably, mitochondrial depolarization triggers a dynamic shift in the interaction pattern of PHB2, promoting its preferential binding to PARL. This interaction drives the release of PGAM5, thereby relieving the inhibition of PINK1 (67). This indicates that autophagosomes can perform dual recognition of damaged mitochondria by different receptor proteins localized to the OMM and IMM. Recent studies have further revealed that the efficient exposure and functionality of PHB2 is dependent on its specific interaction with the mitochondrial outer membrane protein VDAC1: under depolarizing conditions, VDAC1 forms an oligomerization complex with PHB2, and its degradation creates a highly efficient “window of exposure” at the OMM conducive to PHB2-LC3 binding (68). However, whether VDAC1-PHB2 interactions are widely involved in physiological mitochondrial renewal, whether their regulation is cell-type specific, and whether there are other outer membrane proteins that can perform similar functions remain important directions for future research.

### 4.4 Immune signaling receptor

Immune signaling receptors are the most functionally unique class of mitophagy receptors. Unlike most receptors that promote mitophagy, their activation can be induced by pathogens in addition to factors such as hypoxia and energetic stress, thereby promoting mitophagy.

#### 4.4.1 NLRX1

NOD-Like Receptor X1 (NLRX1) is a member of the Nod-like receptor (NLR) family. Its structure comprises a central NACHT domain, a C-terminal LRR, and a unique N-terminal mitochondrial targeting sequence (MTS) that directs the protein through the mitochondrial TOM/TIM complexes into the matrix, where the matrix proteasome cleaves off the first 39 amino acids to form the mature protein (Figure 4). This process was verified by experiments with MTS-deficient mutants, in which MTS-deficient NLRX1 is completely retained in the cytoplasm. Studies reveal that NLRX1 recruits LC3 to mitochondria via the LIR motif within its NACHT domain and promotes damaged mitochondria clearance (69). Although NLRX1 typically localizes to the mitochondrial matrix, mitochondrial depolarization leads to mitochondrial protein import stress (MPIS), causing cytoplasmic NLRX1 retention. Cytoplasmic NLRX1 then interacts with the endoplasmic reticulum protein Ribosome Binding Protein 1 (RRBP1); this complex induces mitophagy by promoting recruitment and lipidation of LC3 around damaged mitochondria. It has also been shown that overexpression of NLRX1 reverses FUNDC1 phosphorylation, upregulates NIPSNAP1/2, and enhances FUNDC1-NIPSNAP1/2 interactions, thereby indirectly activating mitophagy, which explains the multiplexed role of NLRX1 in the maintenance of mitochondrial homeostasis (70). Although NLRX1 has been proposed to regulate multiple cellular processes, including antiviral immunity, apoptosis, reactive oxygen species generation, and mitochondrial metabolism, whether its multifunctional roles integrate through autophagy regulation requires further investigation (71, 72).

### 4.5 Other receptors

This receptor is unique in that it does not mediate typical, wholesale mitophagy but rather operates through an endolysosomal-dependent pathway, functioning only under specific damage conditions.

#### 4.5.1 SAMM50

Sorting and assembly machinery component 50 (SAMM50) is a core component of the *β*-barrel assembly machinery (SAM complex) on the OMM. SAMM50 is responsible for the proper folding and assembly of β-barrel proteins, which are essential for maintaining normal mitochondrial function and morphology. SAMM50 localizes to the OMM and regulates mitochondrial cristae morphology and membrane stability (73). SAMM50 contains several structural domains, of which the POTRA domain and the C-terminal transmembrane domain are its key features (74). SAMM50 mediates basal mitophagy of the SAM and MICOS components by binding to ATG8-family proteins through its N-terminal LIR motif and interacting with p62 (Figure 4) (75). Notably, SAMM50-mediated mitophagy constitutes a partial autophagic process that targets specific mitochondrial components, such as mitochondrial DNA, components of the SAM and MICOS complexes, for lysosomal degradation, thereby facilitating the renewal of specific components within the mitochondria (76, 77). In addition, SAMM50 controls mitophagy by regulating the stability of PINK1, which accumulates at the OMM when SAMM50 is knocked down, thereby promoting mitophagy (78). The case of SAMM50 strongly demonstrates that mitophagy receptors work synergistically with each other to form a complex regulatory network that is both holistic and locally complementary, laying the groundwork for great potential for future research in this field.

## 5 Receptors maintain mitochondrial function via other mechanisms

Beyond mediating mitophagy through diverse mechanisms, mitophagy receptors coordinate with other mitochondrial quality control systems—including mitochondrial dynamics, ion homeostasis, and mitochondrial biogenesis—to sustain cellular energy homeostasis. Mitochondrial dynamics involve continuous fission and fusion processes, through which mitochondria regulate their morphology, abundance, distribution, and functionality to meet cellular physiological demands. Mitochondrial fission produces daughter mitochondria, enabling efficient redistribution to energy-demanding cellular zones. Dysfunctional mitochondria require segregation from the network via fission or alternative mechanisms before mitophagic clearance. The most important significance of mitochondrial fusion is that it promotes mixing between the mitochondrial membrane components and matrix contents. During transient fusion events, mitochondria rapidly exchange contents to optimize morphology and functionality (79). BNIP3 inhibits optic atrophy 1 (Opa1)-mediated mitochondrial fusion while facilitating the recruitment of dynein-related protein 1(Drp1) to mitochondria, thereby promoting damaged mitochondrial breakage and segregation (80, 81). BNIP3 and NIX may indirectly affect the mitochondrial fusion process by regulating the activity of MFN1/2 (82). The phosphorylation state of FUNDC1 modulates its interaction with Drp1 or Opa1 to regulate mitochondrial dynamics (83). Notably, BCL2L13—the sole BCL-2 family protein possessing all four BH domains yet excluded from classical BCL-2 subfamilies—is involved not only in the apoptosis signaling pathway and mitophagy, but also in the regulation of mitochondrial fragmentation. The fission-promoting activity of BCL2L13, which is independent of that of the classic mitochondrial fusion protein Drp1 but requires all four of its BH domains, mutations in any of the BH1-4 domains or transmembrane motifs inhibit this process; however, the underlying mechanism remains unknown (84). In maintaining ionic homeostasis, FUNDC1 interacts with the inositol 1,4,5-trisphosphate receptor type 2 (IP3R2), which stabilizes MAMs and promotes ER Ca2 + transfer to mitochondria and cytoplasm (85). Beyond FTMT-mediated intracellular iron regulation, BNIP3 (86), NIX (87), FUNDC1 (88), and BCL2L13 (57) modulate mitochondrial iron homeostasis by altering mitochondrial morphology or outer membrane permeability. As for mitochondrial biogenesis, recent studies indicate that BNIP3 and NIX can indirectly regulate the expression of PGC-1α and its downstream target gene nuclear respiratory factor 1 (NRF1) through multiple mechanisms, thus affecting mitochondrial biogenesis. Although the precise mechanisms remain unelucidated, BNIP3/NIX, as HIF-1α target genes, likely reinforce HIF-1α-mediated PGC-1α suppression via a negative feedback loop.

## 6 Mitophagy and neurodegenerative diseases

### 6.1 Alzheimer’s disease

**Figure 5:**
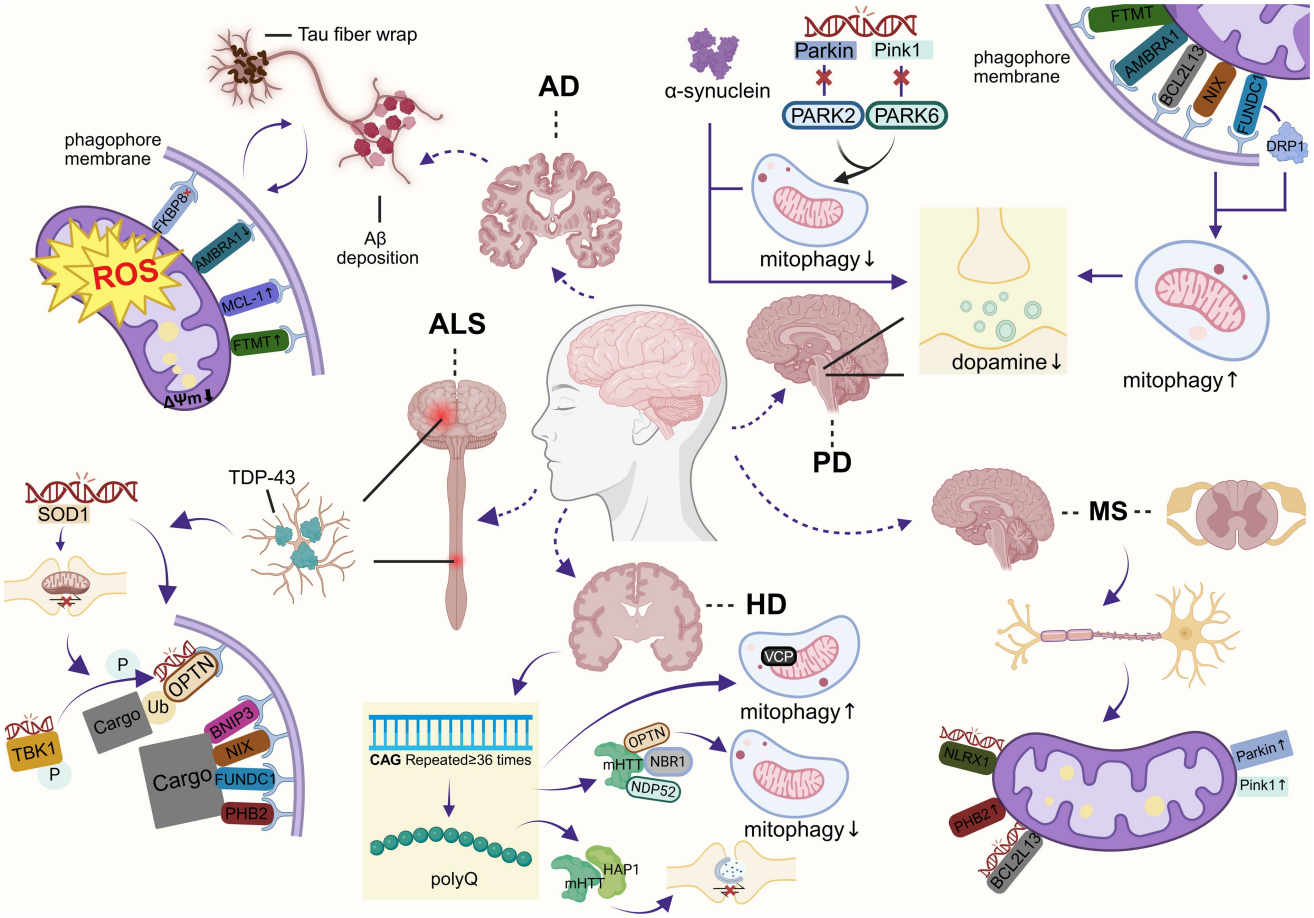
Mechanisms of mitophagy defects in neurodegenerative diseases (created with BioRender.com).

Alzheimer’s disease (AD), the most prevalent neurodegenerative disorder, is characterized by cerebral atrophy in the entorhinal cortex and hippocampus, resulting in synaptic loss, progressive cognitive decline, and mortality. The pathological hallmarks of AD include senile plaques (SPs) formed by extracellular amyloid-*β* protein (Aβ) deposition and accumulation of intracellular hyperphosphorylated microtubule-associated protein tau inclusions, resulting in neurofibrillary tangles (NFTs) that lead to synaptic degeneration, neuron loss, gliosis, and white matter pathology (89). Pathogenesis involves multiple cellular alterations, including dysfunction of mitochondria and synapses, formation and deposition of Aβ and P-Tau. Dysfunction of mitochondria and synapses often occurs early in the AD process. Aβ and P-Tau-induced defects in mitophagy are important changes in AD pathogenesis (90). The main reason for this may be that elevated levels of Aβ and Tau induce ROS production, leading to excessive mitochondrial defects. Studies confirm that restoring mitophagy improves cognitive function in AD mouse models, enhances microglial clearance of Aβ plaques, and reduces neuroinflammation (91). While the association of PINK1-Parkin-induced mitophagy with AD has been extensively demonstrated by several studies in the past, recent scholars have found that receptor-mediated mitophagy does not share the same characteristics in the pathogenesis of AD - the key receptor is directly and precisely hit by the core pathogenic proteins (Figure 5). Most typically, pathologically phosphorylated Tau was shown to specifically interfere with the function of FKBP8, directly inhibiting its ability to initiate mitophagy under oxidative stress (64). Fang et al. observed decreased AMBRA1 expression in AD patient samples and *in vitro* models (91). This suggests that Tau pathology contributes directly to the loss of mitochondria within neurons by “hijacking” specific mitophagy receptors. However, Sepe et al. demonstrated abundant Ambra1 content in neurons in a mouse model of AD (92). This may be due to specific “silencing” of the former by pathologic Tau-P and compensatory elevation of the latter. In addition, MCL-1, as the mitophagy receptor, has been shown to trigger mitophagy and effectively improve cognition in a mouse model of AD through the action of UMI-77 (93, 94). FTMT plays an equally important role in AD. Studies have shown that FTMT expression is significantly increased in brain tissue of AD patients, and that this increase may be related to disturbances in iron metabolism (95). Under oxidative stress conditions, FTMT levels rise and exert neuroprotection by reducing oxidative damage through the ERK/P38 MAPK pathway. However, FTMT overexpression may exacerbate Aβ toxicity. Although the specific mechanism of FTMT in AD remains to be elucidated, this dual ability to regulate iron metabolism and mitigate oxidative stress explains the multidimensional phenomenon of the same receptor in a single cell, rather than a generalized enhancement of “overall mitophagy.” Overall, mitophagy receptors may regulate mitochondrial homeostasis and promote mitophagy in response to mitochondrial stress induced by Aβ and P-Tau via the non-PINK1-Parkin pathway. However, excessive receptor activation drives pathological mitochondrial fission and apoptosis, thereby exacerbating neurodegeneration. Further studies are needed to finely regulate their expression and activity to avoid over-activation and cytotoxicity (Table 1).

**Table 1:** Mitophagy receptors and their functional significance in physiology.

Protein	Mitochondrial localization	Activation signal	Autophagic interactors	Physiological roles	Regulators	Disease	Reference
BNIP3	OMM	Hypoxia	LC3B GABARAPL2	Promote hypoxia-responsive mitophagy	HIF-1α JNK1/2 FBXL4 PPTC7	HD	(134, 135)
NIX	OMM	Hypoxia	GABARAPL1 LC3A LC3B LC3-independent	Mediate selective mitochondrial clearance in hypoxia or erythrocyte maturation	HIF-1α, FBXL4 PPTC7	AD, PD, ALS	(32, 136)
FUNDC1	OMM	Hypoxia	LC3B	Regulate mitochondrial homeostasis by mediating hypoxia-induced mitophagy Involved in metabolic reprogramming and disease	PGAM5 ULK1 MARCH5	PD, ALS	(41, 100, 137, 138)
FTMT	OMM	Hypoxia	LC3	Involved in the regulation of intracellular iron homeostasis, antioxidant defense, and inflammation	HIF-1α NCOA4	AD, PD	(139–141)
AMBRA1	OMM	Mitochondrial stress	LC3 GABARAP	Promote Parkin-mediated mitophagy	CHUK/IKKα, MTOR	AD, PD	(54, 142)
BCL2L13	OMM	Mitochondrial stress	LC3B LC3C GABARAP GABARAP-L	Regulate apoptosis and tumor progression by promoting mitochondrial fission and mitophagy	PRKAA2 AMPKα2 ULK1	PD, MS	(130, 143, 144)
MCL-1	OMM	Oxygen–glucose	LC3A	Regulate apoptosis by various pathways	–	AD	(59, 145)
FKBP8	OMM	Starvation Hypoxia	LC3A	Involved in immunoregulation and mitophagy	–	AD	(146, 147)
PHB2	IMM	Mitochondrial depolarization	LC3B	Regulate PINK1-Parkin-mediated mitophagy Involved in removal of paternal mitochondria	VDAC1	HD, ALS, MS	(114, 131, 148, 149)
NLRX1	OMM	Pathogen Mitochondrial stress	LC3	Regulate the intrinsic immune response and metabolism	RRBP1	MS	(132, 150)
SAMM50	OMM	Mitochondrial depolarization	ATG8, protein GABARAPL1	Fold and assemble β-barrel proteins in the OMM Maintain mitochondrial structure	–	PD	(151)

### 6.2 Parkinson’s disease

Parkinson’s disease (PD) is one of the most prevalent neurodegenerative disorders, featuring dopaminergic neuron degeneration, causing striatal dopamine depletion (96). The pathology is characterized by progressive loss of dopaminergic neurons in the dense portion of the SN and an abnormal accumulation of Lewy bodies containing alpha-synuclein. PD symptoms include motor symptoms such as tremor, bradykinesia, rigidity, and postural instability, as well as non-motor deficits such as dementia, neuropsychiatric disturbances, and autonomic dysfunction. Mitochondrial dysfunction is one of the most important mechanisms in the pathogenesis of PD (Figure 5). It has been found that PD is associated with mutations in *PARK6* and *PARK2* genes, and that abnormalities in Pink1 and Parkin lead to insufficient mitophagy, accumulation of damaged mitochondria, excessive oxidative stress, and energy deficits, which in turn lead to neuronal death and exacerbate PD progression (97). However, *PARK2* gene deletion failed to generalize the PD full phenotypes in mice, indicating the existence of other compensatory mechanisms for mitophagy. Indeed, receptor-mediated mitophagy in PD demonstrates a unique compensatory nature—when the canonical PINK1-Parkin pathway is impaired, multiple mitophagy receptors become specifically activated, forming a robust “molecular backup system.” Recent studies establish NIX-mediated mitophagy as functionally independent of Parkin. In PD patient-derived cells, NIX was found to restore mitophagy by compensating for *PARK6*/*PARK2* mutations (98). In a murine model carrying a glucocerebrosidase *β* (GBA) mutant, which is a genetic risk variant for PD, NIX dimerization was reduced and accompanied by mitophagy dysfunction. Notably, the GBA mutation did not result in Parkin activity and ubiquitination modifications, suggesting that Parkin is less involved in mitophagy defects, and these data indicate that NIX represents an alternative molecular pathway to restore mitophagy in PD (99). FUNDC1, however, exhibits an even more distinctive compensatory mechanism, the Xu team demonstrated that in Drosophila, FUNDC1 significantly ameliorates the phenotype and mitochondrial dysfunction in PINK1 mutant flies. This effect is independent of its LC3-binding domain or the autophagy-related protein ATG7, but is instead mediated by its interaction with dynamin-related Drp1, which provides a potential new target for PD therapy (100). Additionally, evidence indicates that AMBRA1 levels are reduced in patients harboring GBA mutations (99). Furthermore, mitophagy is impaired at both early and late stages in cells expressing the GBA mutant protein. Notably, reductions in AMBRA1 levels have also been observed in pharmacologically induced PD cells and mouse models (101). Similarly, Di Rita A demonstrated that expression of AMBRA1 increased cell viability and reduced the generation of ROS in an *in vitro* model of PD (17). Morales et al. demonstrated that damaged mitochondria accumulate within spheroids formed at the axon terminals of dopaminergic neurons in the SN of rat and mouse models of PD induced by 6-hydroxydopamine and exhibited high levels of AMBRA1 and BCL2L13 immunoreactivity (102). These findings suggest that mitophagy mediated by receptors such as AMBRA1 is critically involved in PD pathogenesis. Another hallmark of PD is the particular accumulation of iron in Lewy bodies. Moreover, iron promotes the aggregation of *α*-synuclein, suggesting that PD and altered iron homeostasis are closely related. Shi et al. demonstrated that FTMT had a significant protective effect against neuronal damage by preventing iron redistribution to maintain iron homeostasis in a 6-hydroxydopamine (6-OHDA)-induced PD model, and that FTMT also exerted its neuroprotective effects by inhibiting oxidative stress, mitochondrial damage, and apoptotic pathways (103).

### 6.3 Amyotrophic lateral sclerosis

Amyotrophic lateral sclerosis (ALS) is a neurodegenerative disorder characterized by concomitant degeneration of both upper and lower motor neurons. This motor neuron loss leads to progressive impairment of motor function, muscle atrophy, and ultimately, paralysis (104). Patients typically succumb to respiratory failure in the late stages of the disease due to paralysis of the respiratory muscles. A pathologic feature of ALS is the cytoplasmic accumulation of TAR DNA-binding protein 43 (TDP-43) in motor neurons, observed in over 95% of patients. In addition, aberrant aggregation of superoxide dismutase 1 (SOD1) and fused in sarcoma protein (FUS) can occur in motor neurons of ALS patients. The exact pathogenesis of ALS has not yet been clarified, and numerous studies have found structural abnormalities, including mitochondrial swelling and significant intracellular mitochondrial aggregation in neurons of patients with sporadic ALS (105). To date, numerous ALS-related genes have been identified, most of which are functionally linked to mitophagy, such as SOD1, OPTN, and TBK1 (Figure 5). Mutant SOD1 blocks the retrograde transport of mitochondria in neurons. In addition, it aggregates and sequesters OPTN, thereby suppressing the formation of mitophagosomes and inhibiting mitophagy flux (106, 107). Moreover, phosphorylation of OPTN at serine 177 by TBK1 kinase is essential for its binding to LC3. ALS-associated mutations impair the OPTN-TBK1 interaction, thereby suppressing autophagosome maturation (108). These findings collectively indicate a significant impairment of mitophagy in ALS, and the accumulation of damaged mitochondria is strongly suggested to play a critical role in its pathophysiology. Rogers et al. observed reduced expression levels of mitophagy-related proteins—including Parkin, PINK1, and BNIP3—in skeletal muscle tissue from ALS patients (109). However, some studies have reported that abnormal activation of AMPK in motor neurons has been proposed as a pathogenic factor in the early stages of ALS (110). Sharma et al. demonstrated that NIX was detected to be upregulated in the cerebrospinal fluid of ALS patients (111). Consistently, upregulation of NIX was documented in astrocytes derived from SOD1 mutant mouse models (112). Additional research identified FUNDC1 expression was down-regulated in the spinal cord of an ALS mouse model, and FUNDC1 overexpression was found to significantly improve motor function in ALS mice by intraspinal injection of AAV9-FUNDC1 into the spinal cord of the mouse model, demonstrating that FUNDC1-mediated mitophagy has a protective effect in ALS, improving motor function, extending survival, and reducing neuronal apoptosis (113). Furthermore, researchers have uncovered a relationship between TDP-43 and PHB2: the level of TDP-43 directly influences the abundance of PHB2, which may represent an alternative compensatory mechanism for mitophagy (114). While mitophagy has been extensively investigated in a variety of neurodegenerative diseases, with considerable consensus on its neuroprotective role, the precise mechanisms underlying its involvement in ALS remain elusive.

### 6.4 Huntington’s disease

Huntington’s disease (HD) is a fatal, inherited neurodegenerative disorder. At the molecular level, expansion of the CAG trinucleotide repeat in exon 1 of the gene encoding huntingtin protein (HTT) results in the N-terminal amplification of 36 or more polyglutamine (polyQ) in the mutant Huntingtin protein (mHTT). This mutant protein accumulates at high levels within neurons of the striatum and cerebral cortex, ultimately leading to neuronal death (115–117). Similar to other neurodegenerative diseases, mitochondrial dysfunction occurs in neurons with HD disease (Figure 5). Current evidence indicates that mitochondrial dysfunction induced by mHTT is a key factor in HD pathogenesis, which is manifested in two main mechanisms: first, the interaction of mHTT with autophagy-related proteins (NBR1, OPTN, and NDP52) disrupts the autophagy pathway (118). Second, mHTT impairs the axonal transport of autophagosomes by affecting huntingtin-associated protein 1 (HAP1), which is more tightly associated with mHTT than with HTT. In the presence of mHTT, impaired autophagosome transport leads to the accumulation of autophagosomes and their cargo, resulting in mitophagy dysfunction (119). HD exhibits a unique phenomenon of bidirectional mitophagy dysregulation. Notably, not only is mitophagy impaired, but its paradoxical overactivation can also drive disease progression. mHTT may recruit valosin-containing protein (VCP) to mitochondria, leading to its accumulation. This induces excessive mitophagy, ultimately resulting in mitochondrial depletion and neuronal death. Furthermore, data from Sassone indicate that BNIP3 accumulates and dimerizes in the mitochondria of human HD muscle cells (120). Separately, Francesca Sassone observed that mutant mHTT failed to induce mitochondrial damage in BNIP3-knockout cells (121). These data suggest that BNIP3 has a potential role in mutant mHTT-induced mitochondrial dysfunction. In addition, inhibition of tissue transglutaminase 2 (TG2) has demonstrated therapeutic benefits in animal models of HD, and PHB2 serves as a substrate for TG2, which may be involved in HD pathophysiological (122, 123). However, further exploration is needed. In summary, the mitophagy pathway in HD does not undergo simple enhancement or suppression, but rather exists in an imbalanced state cohabited by both functional impairment and paradoxical hyperactivation. These alterations drive the accumulation of dysfunctional cellular components, which over time exacerbates disease manifestations.

### 6.5 Multiple sclerosis

Multiple sclerosis (MS) is an immune-mediated neurological disorder characterized by focal demyelination and secondary axonal damage in the central nervous system, representing a leading cause of neurological disability in young adults (124, 125). A substantial body of evidence indicates that mitochondrial dysfunction is closely associated with the pathogenesis and progression of this disease (125, 126) (Figure 5). Pro-inflammatory conditions induce mitochondrial stress, resulting in elevated glucose catabolism and mitophagy. Patergnani et al. observed significantly elevated levels of Parkin and PINK1 in the cerebrospinal fluid of multiple sclerosis patients, which returned to near-baseline levels during disease remission (127). A similar phenomenon was observed in both *in vivo* and *in vitro* MS models, indicating activation of a compensatory mitophagy process (128). This finding has also been confirmed by a cohort study from Japan (129). Variants in the 3’UTR microRNA binding sites of BCL2L13 have been identified in multiple sclerosis, which may alter BCL2L13 protein expression levels and consequently disrupt key processes such as mitophagy and apoptosis (130). Furthermore, researchers have found that elevated PHB2 protein levels in multiple sclerosis may represent a compensatory cellular response to mitochondrial damage, aimed at promoting mitophagy and improving neuronal function (131). Notably, NLRX1—a protein known for regulating the innate immune system—exerts immunosuppressive and anti-inflammatory effects in neurons under autoimmune attack. Rare NLRX1 mutations have been identified in MS patients with a family history (132). To evaluate its therapeutic potential, injection of an NLRX1-LRR fusion protein in a mouse model reduced overall disease severity, decreased T-cell infiltration, and lowered inflammatory cytokine levels in spinal cord tissue (133). This reveals the receptor’s indispensable role in the coordinated regulation of mitophagy and innate immune signaling.

## 7 Receptor-mediated mitophagy as a therapeutic target

Given the crucial role of receptor-mediated mitophagy in neurodegenerative diseases, pharmacological modulation of this pathway may represent an effective therapeutic approach for mitochondria-related disorders. With extensive research, a growing number of compounds (Table 2) have been identified that can restore impaired mitophagy and exert neuroprotective effects in animal or cellular models. Examples include urolithin A (UA), NAD^+^ precursors, among which certain natural products demonstrate particular promise. However, as most studies have been conducted over relatively short timeframes, research on mitophagy modulators remains in its early stages—though this also implies substantial potential for future applications. To achieve therapeutic targeting, such modulators must specifically localize to mitochondria to regulate mitophagy. Yet, most existing agents exhibit limited specificity toward mitophagy. Enhancing their pharmacological precision for mitophagy targets remains a technical challenge. Moreover, given possible differences in etiology and pathological processes across different diseases, there is a need to develop more personalized treatment strategies tailored to the needs of individual patients. In summary, targeting receptor-mediated mitophagy for neurodegenerative diseases is only the beginning. Continued in-depth research is essential to develop highly specific modulators of mitophagy.

**Table 2:** Pharmaceutical modulators of receptor-mediated mitophagy in neurodegenerative diseases.

Pharmaceutical	Mechanistic link to receptor-mediated mitophagy	Health benefits	Disease	Reference
Urolithin A	Increases levels of BCL2L13, AMBRA1	Reduces plaque and soluble Aβ Improves memory function	AD	(91)
NAD precursors: nicotinamide mononucleotide (NMN)	Induces neuronal mitophagy dependent on PINK1, NIX	Reduces Aβ; Alleviates memory and learning deficits	AD	(91)
Kaempferol	Increases levels of PINK1, Parkin, LC3B and AMBRA1	Increases the survival of neurons Abrogates amyloid-β and tau pathologies	AD	(152)
Baicalein	Activates the NIX/BNIP3 pathway	Restored neuronal activity	PD	(153)
Celastrol	Elevating PINK1 and AMBRA1 levels promotes mitophagy	Reduces neuronal death and mitochondrial depolarization	PD	(154)
SBP-1	Target MCL-1 to enhance autophagy	Enhance autophagy to protect neurons	PD	(155)
UMI-77	Induces MCL-1-mediated mitophagy	Reverses molecular and behavioral phenotypes	AD	(93)
Dihydro-resveratrol	Activate BNIP3-dependent mitophagy	Protect microglia from cytotoxicity	AD	(156)
Se-methylselenocys-teine	activate mitophagy flux via the BNIP3/NIX pathway	Increase the cognitive ability	AD	(157)
MCC950	Reduce the overexpression of LC3II ULK1 and AMBRA1	Improve cognitive behavior and reduce anxiety	AD	(158)
Carnosic acid	Ameliorate Aβ-induced mitochondrial imbalance and oxidative stress by upregulating the expression of PHB-1 and PHB-2 genes	Delay age-related paralysis and Aβ deposition	AD	(159)
Rutin	Induce PHB2-mediated mitophagy	Reduce oxidative damage	PD	(160)
Empagliflozin	Maintain mitochondrial integrity through FUNDC1	Prevent cardiac remodeling and improve cardiac function	PD-associated cardiac dysfunction	(161)
Mangiferin	Reverse the expressions of PINK1 Parkin, NIX, BNIP3, FUNDC1	Recover mitochondrial ultrastructure and ATP contents	PD	(162)
Pramipexole	Activate BNIP3-mediated mitophagy	Reduce neuronal injury	PD	(163)
Idebenone	Upregulate BNIP3 expression activating the Parkin/PINK1 mitophagy pathway	Reduce dopaminergic neuronal damage and ameliorate behavioral deficits	PD	(164)
Betulinic acid Hydroxamate	Induce BNIP3 expression	Enhance antioxidant defense in the brain and ameliorate clinical symptoms	HD	(165)

## 8 Conclusions and future perspectives

**Figure 6:**
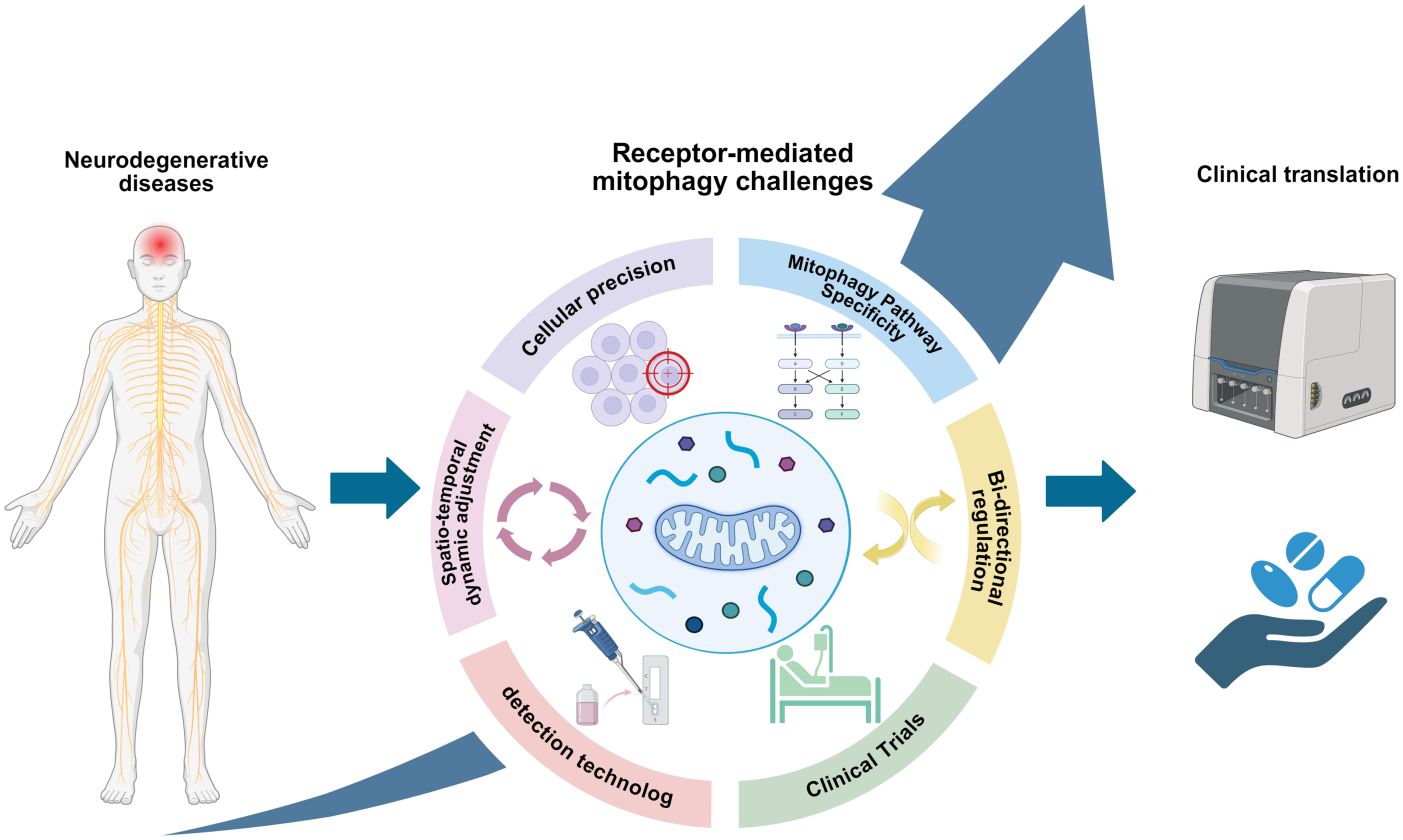
Challenges for future research on receptor-mediated mitophagy in neurodegenerative diseases (created with BioRender.com).

Neurodegenerative diseases (NDDs) still lack effective disease therapies, which highlights a huge gap in our understanding of the complex cellular mechanisms involved. Mitophagy is essential for the control of metabolic homeostasis and the removal of damaged or excess mitochondria, and plays an important role in both the initiation and progression of NDDs. This review elucidates that receptor-mediated mitophagy is not merely a fundamental pathway, but functions as a critical compensatory mechanism when canonical pathways such as PINK1/Parkin are impaired, independently initiating mitochondrial clearance to maintain neuronal survival. This discovery reveals its significant potential as both a biomarker and a therapeutic target. Although we have enumerated numerous synthetic and natural compounds that have been shown to promote such mitophagy, however, translating this potential into practical therapeutic strategies still faces a number of key challenges, such as the temporal and spatial interaction patterns of different mitophagy pathways that are regulated during pathological processes, and how to achieve selective receptor activation while avoiding excessive mitophagy process? And whether they are cell-type specific in the broader physiopathological context have yet to be fully elucidated (Figure 6). These challenges, however, chart a course for future investigation. More extensive and in-depth studies on the intrinsic mechanism of dysregulated mitophagy in NDDs will help to unravel the last veil of NDDs and open new therapeutic avenues for NDDs.
